# Developing resilience throughout the continuum of medical education

**DOI:** 10.1007/s40037-014-0140-1

**Published:** 2014-11-14

**Authors:** Vimmi Passi

**Affiliations:** Masters in Medical Education Masters and CPD Team Warwick Medical School, The University of Warwick, Coventry, CV4 7AL England

The thought provoking paper by Dr Outram on ‘you teach us to listen … but you don’t teach us about suffering—self-care and resilience strategies in medical education,’ highlights the importance for medical educators to consider methods to develop resilience [1]. There has been a recent explosion of interest in teaching and learning resilience in medical education [2–4]. This commentary explores the vital need for medical leaders to develop resilience throughout the continuum of medical education. It is now imperative for medical educators worldwide to collaborate and consider methods to develop resilience for 21st century doctors. We have a duty to support our students and maintain high professional standards of clinical care for our patients.

Resilience is an emotional competence and can be considered as behaviour to be acquired during training [5, p 343]. It consists of cognitive processes that encompass at least four dimensions: self-efficacy; planning; self-control; commitment and perseverance [4, 6]. Interestingly, although there are many definitions of medical professionalism [7], none of the definitions include resilience as a theme. Resilience is a new agenda in education and this commentary explores the importance of developing resilience throughout the continuum of medical education.

The main aim of the medical school curriculum is to develop competent, professional and compassionate doctors. However, studies suggest that mental health worsens after students begin medical school and remains poor throughout training [8]. Life satisfaction has also shown to be reduced during medical school [9]. Potential causes of student distress include adjustment to the medical school environment, ethical conflicts, exposure to death and human suffering, personal life events and educational debt [8]. Therefore, medical educators need to support students to develop resilience throughout the curriculum in their journey to becoming a doctor. This is imperative to avoid the consequences of student distress which include worsening academic performance [10]; loss of empathy [11] and suicide [12].

The journey throughout postgraduate training is exciting but requires both personal and professional resilience. Personal resilience is required in situations as described in Dr Outram’s article regarding ‘managing sad and painful parts of medicine,’ —junior doctors will need to develop skills in ‘breaking bad news,’ patient suffering and end of life care [1]. Professional resilience is required as junior doctors will need to work in different educational environments; there are pressures of postgraduate exams and important decisions to be made regarding their future carer pathway. It is imperative that clinical and educational supervisors support their students and help them develop personal and professional resilience.

The need to develop and maintain resilience continues throughout a doctor’s career. For senior doctors—there are challenges of maintaining effective patient services, work-life balance and workload. Burnout is a frequent phenomenon in experienced practitioners and the most widely used instrument to measure burnout is the Maslach Burnout Inventory (MBI), a questionnaire of 27 items with three subscales [13]. According to Maslach, burnout occurs when three criteria are met: high emotional exhaustion, high depersonalisation and low personal achievement. Medical educators need to conduct further research on burnout to identify the effect of burnout on the mental health of the doctor, the impact on patient care and educational interventions to reduce burn out in doctors.

The challenge for educators is to develop effective, innovative methods within the curriculum to enhance resilience. Dr Outram’s article effectively highlights promising interventions including the importance of a self-care curriculum. However, further research is required on the ways in which resilience can be developed and supported during and after clinical training [4]. Secondly, there is research which suggests that looking at personality traits is more reliable than assessing single measures of resilience or stress. Four personality traits in particular have a strong influence on one’s capacity for developing resilience: self-directedness, cooperativeness, harm avoidance and persistence [14]. Finally, there is no consensus definition of resilience in medical education and this could be valuable for students in understanding the importance of this theme throughout their training.

In summary, developing resilience is an important theme that should be considered a key component in medical professionalism. Developing resilience is particularly important in medicine because of the demanding workloads, societal expectations and government policies [3]. Medical educators need to consider methods to promote resilience and offer support to students and colleagues at all stages of their careers. This is an exciting opportunity and medical leaders worldwide need to embrace the importance of developing a resilient, enthusiastic and passionate future medical workforce.

‘*The practice of medicine is an art, not a trade; a calling, not a business; a calling in which your heart will be exercised equally with your head. Often the best part of your work will have nothing to do with potions and powders, but with the exercise of an influence of the strong upon the weak, of the righteous upon the wicked, of the wise upon the foolish.’ (Sir William Osler 1849*–*1919).*
